# Impact of a telephone triage service for non-critical emergencies in Switzerland: A cross-sectional study

**DOI:** 10.1371/journal.pone.0249287

**Published:** 2021-04-02

**Authors:** Chloé Thierrin, Aurélie Augsburger, Fabrice Dami, Christophe Monney, Philippe Staeger, Carole Clair

**Affiliations:** 1 Department of Ambulatory Care, Center for Primary Care and Public Health (Unisanté), University of Lausanne, Lausanne, Switzerland; 2 Department of Training, Research and Innovation, Center for Primary Care and Public Health (Unisanté), University of Lausanne, Lausanne, Switzerland; 3 Fondation Urgences Santé, Lausanne, Switzerland; 4 Emergency Department, Lausanne University Hospital (CHUV), University of Lausanne, Lausanne, Switzerland; Radboud University Medical Center, NETHERLANDS

## Abstract

**Introduction:**

Telephone triage services (TTS) play an increasing role in the delivery of healthcare. The objective of this study was to characterize the adult users of a TTS for non-critical emergencies, describe the types of advice given and their subsequent observation, and assess the influence of TTS on the use of the healthcare system in a sanitary region of Switzerland.

**Methods:**

Data from a TTS based in the French part of Switzerland were analyzed. This service consists of a medical contact center for non-critical emergencies, with trained nurses available 24/7. A random selection of 2,034 adult calls was performed between July and December 2018. Research students contacted users 2 to 4 weeks after the initial call and assessed sociodemographic and clinical data, as well as the impact of the advice received on the use of the healthcare system.

**Results:**

A sample of 412 users was included in the analyses. The average age was 49.0 (SD 20.4) years; 68.5% were women and 72.8% of Swiss origin. The two main recommendations provided by nurses were to consult the emergency department (ED) (44.6%, n = 184) and to contact a physician on duty (33.2%, n = 137). The majority of users followed the advice given by the nurses (substantial agreement [k = 0.79] with consulting the ED and perfect agreement [k = 0.87] with contacting a physician on duty). We calculated that calling the TTS could decrease the intention to visit the ED by 28.1%.

**Conclusion:**

TTS for non-critical emergencies have the potential to decrease the use of ED services.

## Introduction

During the last decade, Switzerland, like many other Western countries, has observed a large increase in the use of emergency department (ED) services for non-urgent cases, causing significant waiting times and potential delays in the management of more severe cases [1–4]. Telephone triage and advice services play a significant role in the delivery of healthcare [5–7] and are increasingly used to control and curb the growing use of the ED by patients for non-critical emergencies [8].

Health insurance is compulsory for all persons residing in Switzerland and covers all treatments carried out by a physician, including emergency care and first aid. The contribution to costs consists of a fixed annual amount (deductible) and a 10% contribution. Insured persons themselves pay the cost of the medical services they receive until the annual deductible is reached. From then on, they generally pay 10% of the costs exceeding the annual amount. The emergency services in Switzerland are accessible without appointment, either by being referred by a physician or by self-referral.

Throughout Switzerland, the telephone number "144" is assigned for urgent health calls and in the canton of Vaud (western Switzerland), patients can access a telephone triage service (TTS) for non-critical emergencies by using another specific phone number. The TTS is dedicated to the reception, processing and management of non-critical medical calls. The TTS consists of a non-emergency medical center staffed by trained nurses. The nurses are graduates with a minimum of 5 years of experience and ideally a background in acute care. The nurses receive, screen and assess the situation in order to direct the public towards the most appropriate medical solution (involvement of physician on duty—home or office visits, as well as specialists, simple advice while waiting to be able to contact an attending physician, referral to the various care centers (emergencies, medical permanence)). The physician on duty (who is a general practitioner) can only be contacted by the nurses via the TTS. Calls are handled 7-days a week, 24-hours a day [9], and financed by the state government. The TTS receives around 200,000 calls per year, predominantly outside of physicians’ office hours [9].

During the call, the nurses use an internal triage tool. If vital impairment is suspected (disorder of consciousness, circulatory disorder or respiratory disorder), the call is transferred to "144". Further evaluation is carried out using an internal triage tool, which is not computerized. If they have any doubts about triage, the nurses can contact a physician on duty.

Several studies examined telephone triage and showed that one of the main reason for using this service was significant worry [10–12]. The problem of accessibility to care due to working hours and the unavailability of general practitioners were also mentioned as reasons for using TTS by young adults [10]. Previous studies assessed the profile of users of primary care telephone advice services worldwide and showed that sociodemographic characteristics influenced the use of these services. The studies reported that users were more likely to be female and young [12–14] and that those with a low socioeconomic status used the TTS less often than did those with a higher socioeconomic status [12, 15]. The use of TTS has the potential to improve the efficiency of the healthcare system by reducing unnecessary visits to EDs, allowing better allocation of resources.

The main objectives of this study were to characterize the adult users of the TTS, describe the types of advice provided, and assess whether callers follow that advice. The secondary objectives were to assess users’ satisfaction and the potential impact of TTS on the use of emergency services.

## Methods

Ethical approval for the study was obtained from the Commission cantonale d’éthique de la recherche sur l’être humain du canton de Vaud (CER-VD), the competent research ethics committee (CER n°2018–01137).

### Study design

In this cross-sectional study, a research collaborator randomly selected calls each week from all TTS calls made during a 4-month period (July 24 to September 27, 2018, and October 23 to December 17, 2018) by using STATA software (Stata Corp 2015, College Station, Texas, USA). The interruption period was due to administrative problems in the research team. Users were contacted by phone by trained research university students (not necessarily medical students) who collected the data. The students were trained for several hours by the research assistants. During the training sessions, the questionnaire was reviewed and tested, and students learnt how to use the secured software system (REDCap^TM^, Vanderbilt University, Nashville, TN, USA). A research assistant was available at all times for any questions the students might have. During the phone encounter, participants provided oral consent, after which the students recorded their answers on the secured software system (REDCap). The consent was given orally and the answer was transcribed into the Redcap secured form. If participants did not give their consent, the interview was interrupted. This procedure has been approved by the Ethics Commission (CER-VD n°2018–01137). If the answer was negative, the interview was interrupted. For each included participant, a research assistant also retrieved data from the TTS database that had been recorded during the initial call by the nurse. The data from the TTS database were used to know the exact date of the call and the time of the call. These data were retrieved from the registration form and added to the secure folders (REDCap) by the research assistant.

The study protocol is available on the DOI: dx.doi.org/10.17504/protocols.io.bsd3na8n

### Setting and population

The study population consisted of adults (≥ 18 years) living in the canton of Vaud. The inclusion criteria were 1) having good knowledge of French, 2) being able to provide informed consent, and 3) having called the TTS during the study period. The exclusion criteria were 1) relocation outside of the canton of Vaud and 2) the patient having died by the time of the inquiry. We excluded patients who died between the call and the study, because we did not have the resources to collect information on the causes of death and it would have been ethically complex to get participant’s consent.

The sample size calculation was based on the following outcome:: “change of attitude after the TTS call” (dichotomous variable yes/no). An estimated change of 20% in attitude was considered to be clinically relevant with a 95% confidence interval, indicating that a total sample of 430 users was needed to be included.

### Data collection

A research collaborator made a random selection from all eligible calls using the STATA software. Trained students contacted the selected participants 2 to 4 weeks after the initial (index) call. After two failed attempts, the students contacted the next person on their list.

Every user of the TTS included in the study was identified with a unique code number and all identifying information was removed. Data were stored on encrypted and password-protected drives.

We created a questionnaire to assess the sociodemographic and medical characteristics of the participants (S1–S3 Files), the medical reason for the call to the TTS, the satisfaction of the users with the TTS call [16–18], and the potential reduction in ED visits. The questionnaire we used was not an existing validated questionnaire. We constructed it, reviewed it internally with experts from the TTS and tested it with participants similar to the target population. Information on the date and time of the call and the participants’ residence were retrieved from the TTS database, as recorded during the initial call.

We defined the age groups as follows: 18–24 years, 25–59 years, 60–74 years, and ≥75 years in order to allow comparisons with similar studies [5, 16]. The place of residence (city vs. country) was defined according to the definition used by the Swiss Federal Statistical Office [19].

### Data analysis

The usual descriptive statistics were computed for demographic characteristics by using STATA. Proportions and means (with standard deviation) or medians (with interquartile range) were calculated. We analyzed the agreement between variables with the kappa coefficient defined by Landis and Koch [20]: k < 0.00: “poor agreement,” k = 0.00–0.20: “slight agreement,” k = 0.21–0.40: “fair agreement,” k = 0.41–0.60: “moderate agreement,” k = 0.61–0.80: “substantial agreement,” and k = 0.81–1.00: “perfect agreement.” We built univariate and multivariable-adjusted logistic regression models to explore the association between sociodemographic variables and the type of advice received. We chose the variables to be inserted in the model on the basis of a priori knowledge, selecting factors that could influence patient management and that were based on available data.

## Results

**Fig 1:**
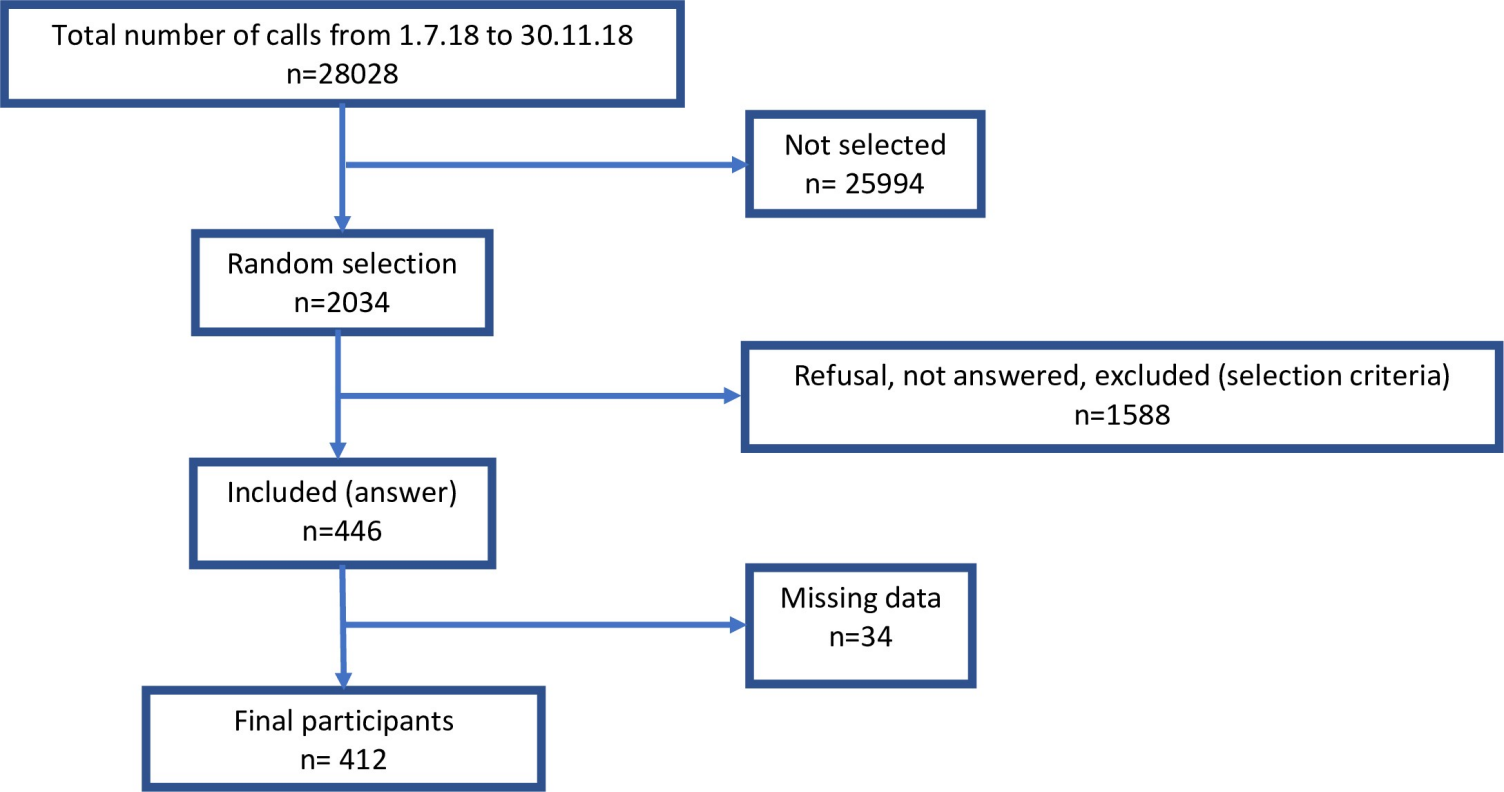
Flowchart of inclusions.

In total, 28,028 calls occurred during the study period and 2,034 calls were randomly selected. Of these, 446 participants answered the phone questionnaire and were included. We further excluded 34 participants because of missing data, which gave a final sample of 412 participants (Fig 1).

### Participant characteristics

When compared with the adult population of the canton of Vaud (n = 643,634) [21], in our study sample there was a higher proportion of women vs. men (68.5% vs. 51.4%, respectively), elderly people (≥ 75 years old) vs. younger people (15.0% vs. 9.8%, respectively), and people of Swiss origin vs. non-Swiss origin (72.8% vs. 66.7%, respectively).

Table 1 presents the sociodemographic and medical characteristics of the participants included in the study. The average age was 49.0 years (SD 20.4, median 45.5 years). Most of the participants were of Swiss origin (72.8%, n = 300). Most participants had completed an apprenticeship and vocational school (41.8%, n = 172), and 27.2% (n = 112) had been to university. A total of 196 participants (47.5%) did not have any professional activity (pension, unemployment). A large majority of participants had a general practitioner (93.9%, n = 387). Most participants reported having a good to very good health status (64.1%).

**Table 1:** Sociodemographic and medical characteristics of the participants.

Category	Characteristics	N = 412	%
Sex	Men	130	31.5
	Women	282	68.5
Age group, years	18–24	42	10.2
	25–59	238	57.8
	60–74	70	17.0
	≥ 75	62	15.0
Country of origin*	Switzerland	300	72.8
	Europe	86	20.9
	Other	26	6.3
Marital status	Single	133	32.3
	Married/Living together	164	39.8
	Widowed	37	9.0
	Divorced/Separated	75	18.2
	Other	3	0.7
Place of residence	City	180	43.7
	Country	230	55.8
	Unknown	2	0.5
Highest level of education	Not completed compulsory school	1	0.2
	Completed compulsory school	74	18.0
	Apprenticeship/vocational school	172	41.8
	High School	47	11.4
	University	112	27.2
	Does not know	3	0.7
	Does not want to answer	3	0.7
Employment rate	Full-time job	128	31.1
	Part-time job	86	20.9
	No activity	196	47.5
	Unknown	2	0.5
Social environment	Living alone	133	32.3
	Living with family or flat sharing	270	65.5
	Medico-social institution	5	1.2
	Does not want to answer	4	1.0
Reported health status	Very good	96	23.3
	Good	168	40.8
	Fairly good	93	22.6
	Bad	48	11.6
	Very bad	5	1.2
	Does not want to answer	2	0.5
Chronic medication	No	232	56.3
	Yes	180	43.7
General practitioner	No	25	6.1
	Yes	387	93.9

*All participants were resident in Switzerland but could have another nationality.

### TTS use

The three main reasons (non-exclusive) reported by participants for calling the TTS were that their general practitioner was not available (36.7%, n = 151), their health problem occurred outside their physician’s office hours (35.0%, n = 144), and that they did not know who to contact (12.1%, n = 50).

Most participants knew of the TTS from their general practitioner (30.8%, n = 127), another health professional (26.2%, n = 108), or the media (18.9%, n = 78). More calls were made during holiday periods (62.8%, n = 259), and the participants called mostly during opening hours (8h-18h) (62.6%, n = 258).

**Fig 2:**
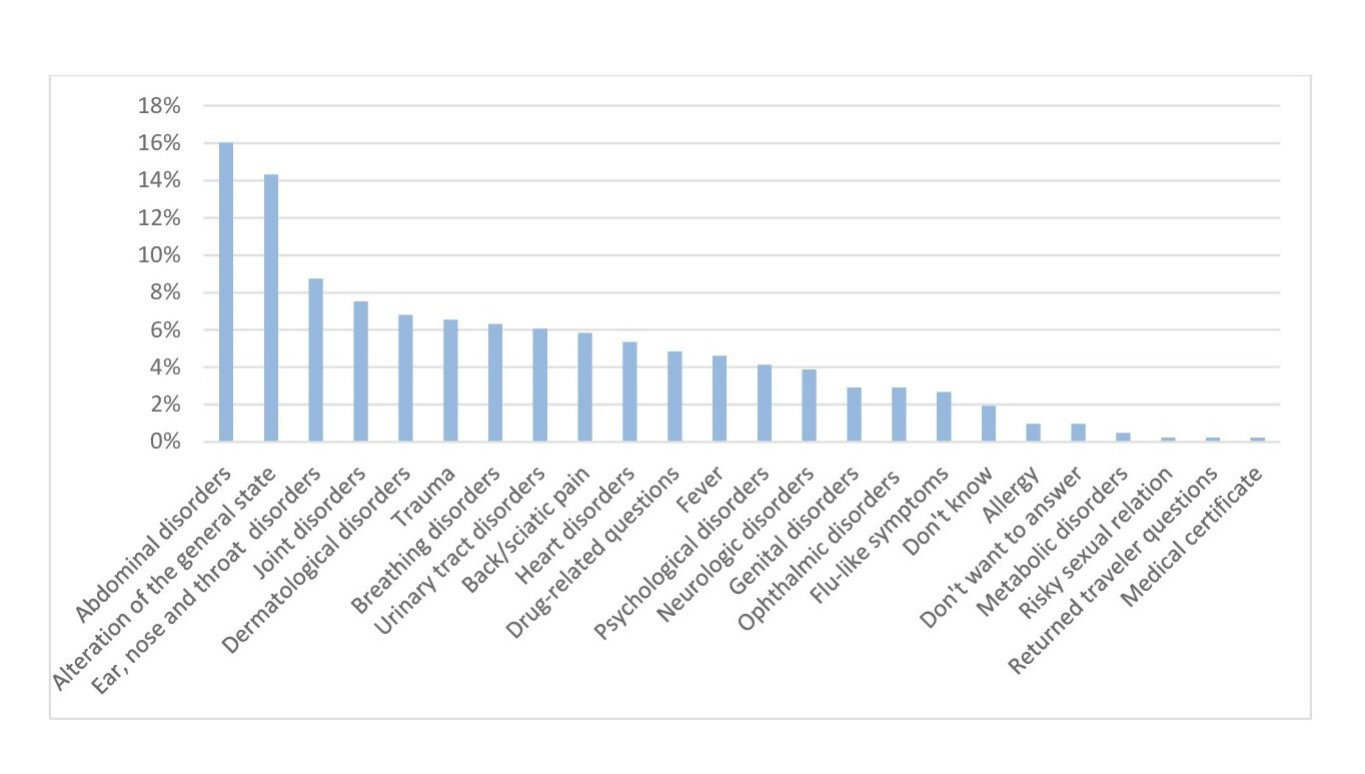
Medical problems reported by telephone triage service users.

The main medical problems reported by users as the reason for calling (more than one reason could be provided) were abdominal disorders (16.0%, n = 66) and alteration of the general state (14.3%, n = 59). The entire list of medical problems reported is shown in Fig 2.

### Impact of the TTS

**Fig 3:**
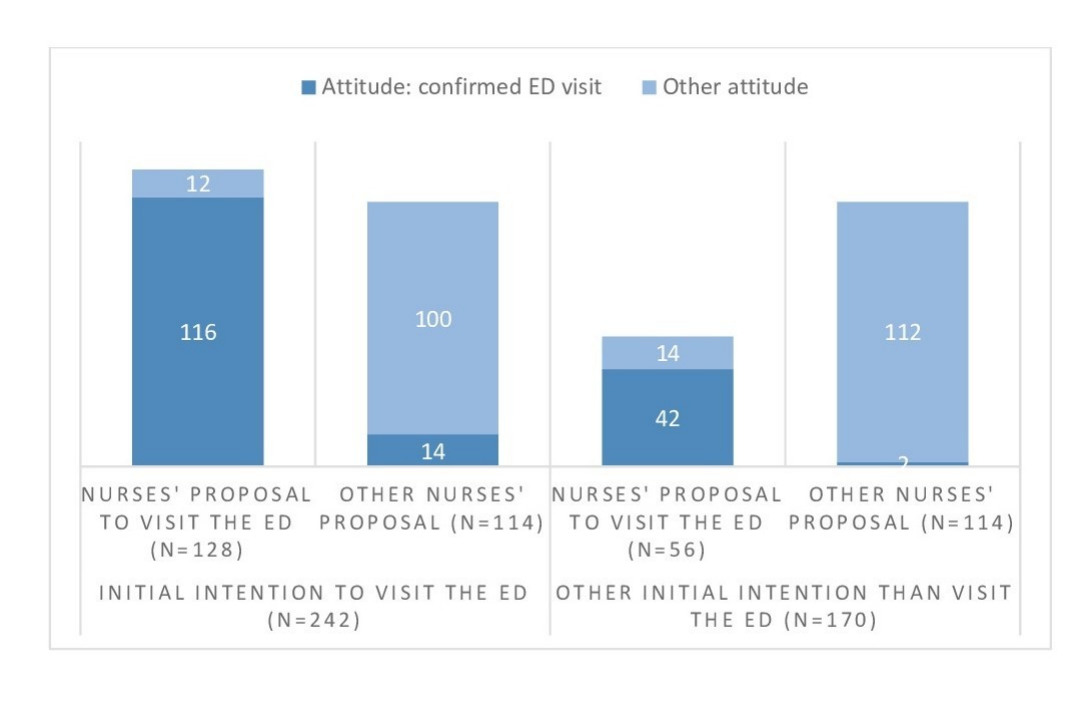
Distribution between proposal made by telephone triage service (TTS) staff and attitude of TTS users according to their primary intention to visit the emergency department (ED) vs. other primary intentions.

Among the participants included, without the possibility of calling the TTS, most of the participants (58.7%, n = 242) would have consulted the ED in a hospital or in a walk-in clinic. Among them, 114 (47.1%) received another proposal through the TTS, and 100 (87.7%) followed the advice given, while 14 (12.3%) still consulted the ED. Among the 170 patients that did not intended to go to the ED, TTS nurses proposed to 42 patients (24,7%) to consult the ED. (Fig 3)

In total, the TTS nurses proposed consulting the ED for 44.6% of the participants (n = 184), contacting a physician on duty (residency, practice, telephone) for 33.2% of them (n = 137), proposed advice and self-care provision to 18.7% (n = 77), consulting their general practitioner to 3.6% (n = 15) and consulting a specialist physician to 2.7% (n = 11). Six patients (1.5%), could not remember the advice given. The total is higher than the number of patients, as nurses sometimes gave several alternative advices. We estimated that calling the TTS contributed to a decrease of 28.1% in the intention to visit immediately the ED among this specific population (242 participants wanted to visit the ED before the call and 174 visited the ED after the call).

Agreement between the intentions of the TTS users, the nurses’ advice, and the users’ final decision are shown in Table 2. There was a slight agreement (k = 0.18) between the intention of users to consult the ED and the advice provided by the nurses to consult the ED, suggesting that the TTS nurses proposed an alternative to consultation with the ED.

**Table 2:** Agreement between the users’ intention before the call, the proposal made by the TTS nurse, and the users’ attitude after the call.

	Kappa Intention—Proposal	Kappa Proposal—Attitude	Kappa Intention—Attitude
Consultation in the ED (hospital or walk-in clinic)	0.18	0.79	0.26
Contact with a physician on duty (home, practice, telephone)		0.87	
Consultation with their general practitioner	0.03	0.40	0.11
Consultation with another healthcare professional	0.07	0.36	0.12
Advice, self-care provision		0.69	

Landis and Koch [20] defined the kappa coefficient as follows: k < 0.00: “poor agreement,” k = 0.00–0.20: “slight agreement,” k = 0.21–0.40: “fair agreement,” k = 0.41–0.60: “moderate agreement,” k = 0.61–0.80: “substantial agreement,” and k = 0.81–1.00: “perfect agreement.” ED: Emergency Department.

Overall, users followed the advice provided by the TTS nurses, as illustrated by a substantial to perfect agreement with them regarding consultation with the ED (k = 0.79), contact with the physician on duty (k = 0.87), and waiting without consultation/nurses’ advice (k = 0.69). The users were less likely to follow the advice to consult their general practitioner (k = 0.40) and to consult another healthcare professional (k = 0.36).

The users changed their attitude after the call to the TTS, as shown by the fair agreement (k = 0.26) between the intention of the users to consult the ED and consultation with the ED after the call.

### Variables influencing management

In the multivariable-adjusted model, older people (≥75 years) were 72% less likely to receive advice to go to the ED (odds ratio [OR] 0.28, 95% confidence interval [CI] 0.12–0.67) and were almost 6 times more likely to be referred to a physician on duty (residency, practice, telephone) (OR 5.74, 95% CI 2.16–15.22) compared with younger users (18–24 years old) (Table 3).

**Table 3:** Variables predicting the probability of receiving advice to consult the emergency department or to consult a physician on duty (multivariate logistic regression model).

	Advice to consult the emergency department		Advice to consult a physician on duty	
Variable	OR	95% CI	OR	95% CI
Age, years				
18–24 (ref.)	Ref.	Ref.	Ref.	Ref.
25–59	0.83	0.42–1.64	2.24	0.92–5.41
60–74	0.74	0.34–1.63	**3.03**	**1.15–7.96**
>75	**0.28**	**0.12–0.67**	**5.74**	**2.16–15.22**
Sex (men vs. women)	1.23	0.79–1.91	0.78	0.49–1.24
Country of origin (foreigners vs. Swiss)	0.96	0.87–1.06	0.98	0.87–1.10
Medical problem reported				
Abdominal disorders	1.70	0.81–3.56	0.81	0.36–1.8
Alteration of the general state	0.86	0.43–1.73	1.79	0.89–3.61
ENT disorders	0.58	0.23–1.42	**2.95**	**1.21–7.16**
Joint disorders	**2.73**	**1.10–6.76**	0.52	0.18–1.48
Dermatological disorders	1.29	0.51–3.23	0.61	0.20–1.80
Other	1.15	0.63–2.09	1.17	0.62–2.20

ref: reference, ENT: ear-nose-throat, OR: odds ratio, CI: confidence interval. Bold numbers represent significant results.

Participants who reported joint disorders were more likely to be referred to the ED (OR 2.73, 95%, CI 1.10–6.76) than were other users, and participants who reported ENT disorders were more likely to be referred to the physician on duty (OR 2.95, 95% CI 1.21–7.16). For other medical problems, the differences were not statistically significant (Table 3). The participants’ sex and country of origin did not influence the proposals made by the TTS nurses.

### User satisfaction

Overall, users were satisfied with the TTS, as shown from the mean score of 8.56 out of 10 (SD 1.8). The feeling of having been listened to was scored as 8.77 (SD 1.7), the belief that the TTS met their expectations was 8.55 (SD 2.0), and professionalism was graded as 8.85 (SD 1.7).

## Discussion

### Main findings

#### Potential impact of the TTS

Without the possibility of calling the TTS, most of the participants in our study (58.7%) would have consulted the ED (hospital or a walk-in clinic). In a study by Li et al. [22], they analyzed medication-related calls to a TTS in Australia by 23,254 participants and found that most participants reported that they would have consulted their general practitioner or another healthcare professional if a TTS was not available; only 5% of them had planned to visit the ED. The difference between this study and ours can be explained by the differences in healthcare systems, in Switzerland there is easy access to emergency services because of the wide offer.

The nurses proposed that most participants consult the ED. The second major proposal was that the participants contact the physician on duty. This result is probably a result of the lack of other services after office hours and the need for further examination. Li et al. [22] reported that the first proposal was self-care and advice, probably because their study focused only on medication-related calls and there was no option to visit the ED. Mulcahy et al. [23] analyzed nurses’ triage performance in a center in Ireland, where general practitioners also responded to TTS calls, and reported that most problems were solved by telephone advice and only 14.3% of participants were referred to the ED. In the study by Van Ierland et al. [24] in the Netherlands, which examined the validity of a triage system, after the TTS call, 35% of patients received only a telephone consultation and 65% had a consultation with the physician on duty in the office or at home. However, the results are difficult to compare as the telephone consultation or the consultation with the physician on duty could lead to a consultation in the ED.

In our study, after the call with the TTS, most of the participants followed the nurses’ advice. This result has also been reported in other studies in Switzerland [25] and in other countries [26].

The users in the present study changed their attitude after the call to the TTS, as shown by the fair agreement between the intention of the users to consult the ED and the consultation with the ED after the call. In our survey, 68 of 242 users decided not to visit the ED after the call, resulting in a potential reduction in the intention to visit the ED of about 28%.

TTS offers an alternative to consultation in the ED, for example, with an appointment with the physician on duty. On a larger scale, the decrease in ED visits might allow shorter waiting times in the ED and may reduce the delay in the management of severe cases. Several studies in Ireland and Sweden have demonstrated that telephone advice services and case management help to decrease the number of visits to the ED [23, 27] and potentially decrease health costs.

#### Participant characteristics

Women were more likely than men to use the TTS in this study (68.5%), which has also been observed in other studies [7, 10–15, 22, 28].

This study also showed that, although they were less likely to participate, elderly people were more likely to use the TTS than were younger age populations, in contradiction with the results of other studies [13–15]. This service has existed in our sanitary region since 1961 and is well-known by the population, including elderly people who are also the most frequent users of the primary healthcare system. The difference in results could be explained by the better knowledge and access to this service by elderly people in our region, the information being provided mainly by healthcare providers.

This study also highlighted the fact that people of Swiss origin were more likely to use the service. This could be explained by the language barrier that some non-Swiss people might encounter. The non-Swiss population might also be less informed about the TTS. This is also the case in Denmark [10, 12] and the Netherlands [11], where native Danes and Dutch people use TTS more frequently. Some studies [13, 15] have assessed TTS access by ethnicity and shown that white people were more likely to call compared with other ethnic groups.

The three main reasons reported by participants for calling the TTS were that their general practitioner was not available, their health problem occurred outside their physician’s office hours, and they did not know who to contact. The vast majority of TTS users had a general practitioner (93.9%), a proportion that was larger than in the State of Vaud (about 80%). This is an interesting finding, suggesting that participants did not use the TTS not because they did not have a general practitioner, but rather because he or she was not available. Having a general practitioner might also increase the likelihood of using a TTS because general practitioners are the main source of information about it (30.8%). These results are in congruence with the study by Moth et al. [10] conducted in Denmark, in which young people (18–39 years) reported that it was difficult to have contact with their general practitioner. On the other hand, in the study by Keizer et al. in the Netherlands [11], which focused on participants whose call was considered non-critical, this problem was less mentioned by participants whose main reason for calling was that they were worried about their health and the study by Huibers et al. [12], shows similar results for patients who contact the TTS. This motive is less evident in our study.

Finally, most of the participants reported having a fairly good to very good health status (86.7%). This result is similar to that of other studies [10, 12, 13], although health status was not categorized identically. Thus, TTS might target people with a relatively good auto-evaluated baseline health status, whereas the primary intention of people with poorer health might be to use other services such as the ED, and chronically ill people who know themselves well are likely to have other resources (specialists, etc.).

#### Variables associated with management

We demonstrated that elderly people were less likely to be referred to the ED than were younger users and were more likely to be referred to a physician on duty. This can be explained by the fact that the TTS proposes home visits by physicians on duty, which could be more acceptable and adequate for elderly people who are less mobile than younger people. This system has the potential to lower health costs by reducing unnecessary ambulance transfers. The sex and country of origin of the users did not influence the proposals made by the nurses. This is an interesting and reassuring finding, as it suggests that management by TTS nurses is not subject to gender- or ethnicity-based stereotypes.

#### User satisfaction

Evaluation of user satisfaction of TTS is difficult to assess because of a lack of rigorous methods and scales [17, 29]. However, most studies that evaluated user satisfaction demonstrated that it was high overall [13, 16–18, 28, 30]. The participants appreciated being able to contact medical personnel outside of their general practitioner’s office hours [13, 16], quick and easy access to care, and less of a need to travel [18, 30].

### Strengths and limitations

This is the first survey to assess the sociodemographic data of TTS users in the State of Vaud, the reason for the call, the advice given by the TTS, and the fate of patients after the call. We used hetero-administered questionnaires and collected good quality data among more than 400 participants. One of the strengths of this study is that it investigates a TTS, which is one of the most widespread systems for after-hours primary care in Europe (general practitioner cooperative) [31].

One limitation of the study is that users who were not fluent in French were excluded, which induced a selection bias. There was also a response bias, as a higher proportion of the participants were women and a lower proportion were elderly people. The profile of all users (n = 28,028) could not be compared with the sample selected for our study. It was indeed not possible and unethical to have more information. During the calls, only some information were listed routinely: the date of the call, the time of the call, the reason of the call and the proposal made by the nurses. Furthermore, it would not have been ethical to publish more data on all TTS users without having obtained their consent. The study was conducted over summer and fall periods, including several weeks of school holidays. During these periods, a higher proportion of general practitioners might be absent. This may have influenced TTS use and the data may therefore not be representative of other non-holidays periods. The duration of the study should have been one year to account for seasonality. Unfortunately, we did not have the resources to conduct this study over one year. Nevertheless, it covers 3 seasons (summer, autumn and early winter). Despite the short recall period (2–4 weeks), a recall bias is still possible. Finally, we did not have information on the patients’ outcome. It is not possible, from our data, to evaluate whether the triage advice was justified on the basis of the patient’s initial report of their health problem.

### Implications for research and/or practice

In conclusion, in most cases, users of the TTS in the State of Vaud followed the advice given by the staff members. We calculated that the use of TTS might decrease the intention of this population to visit the ED by 28.1%. If we extrapolate these results to the number of calls per year to the TTS in the canton of Vaud (200,000), TTS could potentially save around 30,000 ED consultations per year in our sanitary region. The results of the study were sent to the TTS and then to the health department of the canton of Vaud with the aim of obtaining data to improve the health system.

## Supporting information

S1 File Questionnaire original French 1. (DOCX)

S2 File Questionnaire original French 2. (DOCX)

S3 File. Questionnaire translated English (DOCX)
