# Supplementary material for: Impact of a telephone triage service for non-critical emergencies in Switzerland: A cross-sectional study
Source: PLoS One. 2021 Apr 2;16(4):e0249287. doi: 10.1371/journal.pone.0249287 (PMC8018644; doi:10.1371/journal.pone.0249287)
Supplement: S1 File — (DOCX) [file pone.0249287.s001.docx]

**CTMG_tel**

*Faire remplir le questionnaire une fois que la feuille de consentement a été approuvée.*

*Merci !*

*_________________________________________________________________________________*

*Numéro d'identification*

*_________________________________________________________________________________*

*Date du jour*

*_________________________________________________________________________________*

*(Info sur appel à la CTMG)*

*Lire la feuille d'information et voir si accepte de participer ou pas*

Nous allons commencer par vous poser des questions sur votre dernier appel la Centrale Téléphonique des Médecins de Garde

*_________________________________________________________________________________*

Consentement de participation donné oralement

- Oui
- Non

*_________________________________________________________________________________*

Pour quelles raisons avez-vous appelé la centrale?

- Baisse de l'état général/je suis pas bien, malaise/perte de connaissance
- Etat grippal/crève/grippe
- Fièvre/température
- Tension trop haute ou trop basse, douleurs dans la poitrine, palpitations/cœur qui bat vite, œdème/jambes gonfles (Problème cardiovasculaire)
- Toux, essoufflement/peine à respirer, bronchite, crachat de sang (Problème respiratoire)
- Nez qui coule, saigne du nez, sinusite, douleurs des oreilles, sifflement dans les oreilles, écoulement des oreilles, diminution de l'ouïe, mal à la gorge, boule dans le cou/adénopathie, vertiges (Problème ORL)
- Mal au ventre, brûlures d'estomac, diarrhées, constipation, sang dans les selles, nausées, vomissements, perte d'appétit, perte/prise de poids, hémorroïdes (Problème abdominal)
- Lumbago/mal au dos, sciatique
- Fracture, entorse, rougeur ou douleur d'une articulation, arthrose (Problème des os ou des articulations autres que le dos)
- Rougeur, brûlure, bouton/éruption, démangeaisons, piqure de tique ou d'insecte, morsure (Problème cutané)
- Hyperglycémie ou hypoglycémie, diabète (Problème métabolique)
- Œil rouge, douloureux, qui coule, démange, vison trouble ou diminuée, corps étranger (Problème oculaire)
- Troubles du sommeil, anxiété, stress, tristesse, idées suicidaires, tentative de suicide (médicamenteux ou autre) (Problème psychologique)
- Migraines/mal à la tête, vertiges, tremblements, perte de force ou de sensibilité d'un membre (Problème neurologique)
- Brûlure en faisant pipi, besoin d'aller uriner plus souvent, sang dans les urines, perte d'urine (Problème urinaire)
- Retard de règle, risque de grossesse, perte de sang, démangeaisons, écoulement anormal/perte (Problème génital)
- Rapport sexuel à risque
- Problème au retour de voyage
- Traumatisme, accident, plaies
- Problème de médicament
- Réaction allergique
- Autre
- Je ne sais plus
- Je ne souhaite pas répondre

*_________________________________________________________________________________*

Veuillez préciser

*_________________________________________________________________________________*

Si vous n'aviez pas pu appeler la Centrale téléphonique des médecins de garde, qu'est-ce que vous auriez fait?

- Rien
- J'aurais pris des médicaments par moi-même sans consulter
- J'aurais demandé un avis à un proche (ami, famille)
- J'aurais appelé mon médecin généralise dès que possible
- J'aurais consulté mon médecin généraliste ou un autre médecin sur rendez-vous dès que possible
- J'aurais consulté aux urgences d'un hôpital
- J'aurais consulté aux urgences d'une permanence
- Je me serais renseigné sur internet (ou autre)
- J'aurais demandé un avis à un autre professionnel de la santé (ostéopathe, naturopathe, homéopathe)
- J'aurais demandé un avis en pharmacie
- Autre
- Je ne sais pas
- Je ne souhaite pas répondre

*_________________________________________________________________________________*

Précisez

*_________________________________________________________________________________*

Lors de votre appel, quelle suite de prise en charge vous a-t-on proposée?

- Consulter aux urgences d'un hôpital
- Consulter dans une permanence
- Prise de rendez-vous à la maison de la garde
- Mise en contact avec le médecin de garde
- Prendre contact avec mon médecin généraliste pour un rendez-vous dès que possible
- Attendre
- Transfert de l'appel au médecin de garde pour avis sur suite de prise en charge
- Conseils (surveillance, contrôle de la température, prise de traitement)
- Recourir à un autre service
- Autre
- Je ne sais pas
- Je ne souhaite pas répondre

*_________________________________________________________________________________*

Précisez

*_________________________________________________________________________________*

Suite à votre appel à la centrale, qu'avez-vous fait?

- J'ai appelé le 144 pour une ambulance
- J'ai consulté aux urgences d'un hôpital
- J'ai été dans une permanence
- J'ai été au rendez-vous à la maison de la garde
- J'ai eu un contact avec le médecin de garde pour une visite au cabinet
- J'ai eu un contact avec le médecin de garde pour une visite à domicile
- J'ai pris contact avec mon médecin généraliste pour un rendez-vous dès que possible
- J'ai attendu
- J'ai suivi le conseil (surveillance etc.)
- J'ai rappelé la Centrale
- Autre
- Je ne sais pas
- Je ne souhaite pas répondre

*_________________________________________________________________________________*Précisez

*_________________________________________________________________________________*

Pour quelle raison avez-vous appelé la centrale téléphonique des médecins de garde plutôt qu'un autre service?

- J'aime appeler la centrale
- C'était hors des heures d'ouverture
- J'étais trop malade pour sortir
- J'ai suivi les conseils d'ami/famille
- Je ne savais pas qui contacter
- C'était urgent/ je ne voulais pas attendre
- Je ne voulais pas embêter mon médecin généraliste
- J'étais anxieux/se
- Mon médecin traitant n'était pas disponible
- Je n'ai pas de médecin généraliste
- Sur conseil de mon assurance maladie
- Autre
- Je ne sais pas
- Je ne souhaite pas répondre

*_________________________________________________________________________________*

Veuillez préciser

*_________________________________________________________________________________*

Comment connaissez-vous la centrale téléphonique des médecins?

- Via une connaissance (ami, famille, proche)
- Par mon médecin généraliste
- Par mon pharmacien
- Par un autre professionnel de la santé
- Par les médias
- Autre
- Je ne sais pas
- Je ne souhaite pas répondre

*_________________________________________________________________________________*

Veuillez précisez

*_________________________________________________________________________________*

Était-ce votre premier appel à la centrale téléphonique des médecins de garde?

- Oui
- Non

*_________________________________________________________________________________*

Si non, environ combien d'appels avez-vous fait pour vous-même dans les 12 derniers mois?

- c'est le premier appel au cours des 12 derniers mois
- 2-5
- 5-10
- plus que 10

*_________________________________________________________________________________*

**Nous allons vous posez quelques questions sur votre satisfaction suite à l'appel**

*_________________________________________________________________________________*

Globalement, sur une échelle de zéro à dix, comment évalueriez-vous votre satisfaction de l'appel à la Centrale?

(0=pas du tout satisfait, 10=très satisfait)

Sur une échelle de zéro à dix, à quel point vous êtes-vous senti écouté par la personne qui a répondu à votre appel?

(0=pas du tout écouté, 10=tout à fait écouté)

Toujours concernant votre appel, sur une échelle de zéro à dix, à quel point diriez-vous que l'appel à répondu à vos attentes?

(0=pas du tout, 10=tout à fait)

Sur une échelle de zéro à dix, à combien jugeriez-vous le professionnalisme de la prise en charge de votre appel?

(0=pas du tout professionnel, 10=très professionnel)

*_________________________________________________________________________________*

Combien de temps selon vous avez-vous attendu avant qu'on vous réponde ?

- moins d'une minute
- 1 à 5 minutes
- 6 à 10 minutes
- 11 à 15 minutes
- 16 à 30 minutes
- 31 minutes à 1 heure
- plus d'une heure
- je ne sais pas
- je ne souhaite pas répondre

*_________________________________________________________________________________*

**Démographie**

*_________________________________________________________________________________*

Nous allons maintenant poser des questions générales sur votre personne

*_________________________________________________________________________________*

Quel est votre sexe?

- femme
- homme
- autre

*_________________________________________________________________________________*

Quel âge avez-vous? (en année)

*_________________________________________________________________________________*

Quel est votre état civil?

- Célibataire
- Marié-e
- Veuf / veuve
- Divorcé-e
- Séparé-e
- Lié-e par partenariat enregistré/ concubinage
- Partenariat enregistré dissous
- Autre

*_________________________________________________________________________________*

Dans quel pays êtes-vous né(e)?

- Suisse
- Europe de l'Est (Russie, Ukraine, Biélorussie)
- Europe de l'Ouest (Royaume Uni, France, Irlande, Belgique, Pays-Bas, Luxembourg)
- Europe du Nord (Norvège, Danemark, Islande, Suède, Finlande, Estonie)
- Europe du Sud (Espagne, Portugal, Italie, Saint-Martin, Croatie, Grèce, Monténégro, Bulgarie, Bosnie, Serbie, Albanie, Macédoine, Kosovo)
- Amérique du Nord
- Amérique du Sud
- Afrique du Nord
- Afrique du Sud
- Asie
- Autre

*_________________________________________________________________________________*

Veuillez précisez

*_________________________________________________________________________________*

Depuis combien de temps vivez-vous en Suisse?

- moins d'une année
- plus d'une année
- je ne souhaite pas répondre

*_________________________________________________________________________________*

Veuillez préciser

*_________________________________________________________________________________*

Quel passeport ou permis de séjour avez-vous?

- passeport suisse
- permis de séjour
- touriste ou étudiant
- autre
- je ne sais pas
- je ne souhaite pas répondre

*_________________________________________________________________________________*

Quel est le niveau scolaire le plus élevé que vous avez achevé?

- n'a pas achevé ou fréquente encore l'école obligatoire
- école obligatoire achevée
- apprentissage, formation professionnelle
- maturité fédérale ou lycée
- haute école spécialisée et université
- autre
- je ne sais pas
- je ne souhaite pas répondre

*_________________________________________________________________________________*

Veuillez préciser

*_________________________________________________________________________________*

Quel âge aviez-vous lorsque vous avez quitté l'école?

*_________________________________________________________________________________*

Avez-vous des enfants?

- Oui
- Non

*_________________________________________________________________________________*

Si oui, combien?

*_________________________________________________________________________________*

Sont-ils à votre charge?

- oui tous
- oui une partie
- non

*_________________________________________________________________________________*

Veuillez indiquer l'âge de l'enfant?

(en année, si à moins d'1 an mettre: 0.1 pour 1 mois, 0.2 etc jusqu'à 0.11 pour 11 mois)

*_________________________________________________________________________________*

Veuillez indiquer l'âge de l'enfant?

*_________________________________________________________________________________*

Veuillez indiquer l'âge de l'enfant?

*_________________________________________________________________________________*

Veuillez indiquer l'âge de l'enfant?

*_________________________________________________________________________________*

Veuillez indiquer l'âge de l'enfant?

*_________________________________________________________________________________*

Veuillez indiquer l'âge de l'enfant?

*_________________________________________________________________________________*

Veuillez indiquer l'âge de l'enfant?

*_________________________________________________________________________________*

Combien d'entre eux, vivent à la maison ?

*_________________________________________________________________________________*

Quelle est la structure de votre ménage et de votre lieu de vie?

- Vit seul(e)
- Vit avec les parents ou un seul parent
- Vit en couple sans enfant
- Vit en couple avec enfant(s)
- Parent élevant seul un ou des enfant(s)
- En colocation avec des amis, connaissances
- Dans un foyer d'étudiants, internat
- Dans une institution sociale (foyer social, établissement médico-social)
- Sans domicile
- Autre
- Je ne souhaite pas répondre

*_________________________________________________________________________________*

Avez-vous actuellement une activité professionnelle?

- non
- oui à temps partiel
- oui à temps plein

*_________________________________________________________________________________*

Êtes-vous actuellement au bénéfice d'une rente?

- Non
- Indemnité de chômage
- Aide sociale
- Rente AI
- Rente AVS
- Autre
- Je ne sais pas
- Je ne souhaite pas répondre

*_________________________________________________________________________________*

Précisez

*_________________________________________________________________________________*

**Etat de santé**

*_________________________________________________________________________________*

Nous souhaiterions à présent vous posez des questions sur votre état de santé

*_________________________________________________________________________________*

Comment évaluez-vous votre état de santé en général?

- très bon
- bon
- assez bon
- mauvais
- très mauvais
- je ne souhaite pas répondre

*_________________________________________________________________________________*

Avez-vous un médecin traitant?

- Oui
- Non

*_________________________________________________________________________________*

Si oui, où?

- dans un cabinet
- dans une permanence/policlinique

*_________________________________________________________________________________*

Veuillez indiquer si vous souffrez actuellement d'une des maladies suivantes:

- Aucune
- Hypertension artérielle
- Problème cardiaque
- Diabète
- Bronchite chronique (BPCO)
- Arthrose/problèmes articulaires
- Douleurs autres
- Cancer
- Autre
- Je ne souhaite pas répondre

*_________________________________________________________________________________*

Précisez

*_________________________________________________________________________________*

Prenez-vous actuellement un traitement médicamenteux tous les jours depuis plusieurs mois?

*--> (on cherche à savoir si traitement chronique, exclusion des compléments alimentaires, vitamines, homéopathie, gouttes de Bach et autre médecine complémentaire)*

*--> pour les femmes pensez à demander si contraception*

- Oui
- Non

*_________________________________________________________________________________*

En moyenne, combien de traitement/médicament prenez-vous chaque jour?

*(nous cherchons à savoir le nombre de médicaments différents par jour et pas si prend 1 médicament plusieurs fois par jour)*

*_________________________________________________________________________________*

Au cours des 12 derniers mois, avez-vous consulté au moins une fois un des professionnels de la santé suivants? Si oui, lequel ou lesquels?

- Médecin de famille, généraliste, interniste
- Médecin spécialiste
- Autre professionnel de la santé (infirmier-ère en diabétologie, diététicien-ne, podologue, psychologue, physio)
- Service des Urgences d'un hôpital
- Policlinique/permanence
- Autre
- Aucun

*_________________________________________________________________________________*

Combien de fois sur les 12 derniers mois?

*_________________________________________________________________________________*

Veuillez préciser

*_________________________________________________________________________________*

Vous arrive-t-il d'utiliser internet pour vous renseigner sur des problèmes médicaux?

- oui
- non
- je ne souhaite pas répondre

*_________________________________________________________________________________*

Si oui, à quelle fréquence utilisez-vous internet pour des informations sur la santé?

- Jamais
- Presque jamais (moins d'une fois par année)
- Parfois (1-3x par année)
- Souvent (tous les mois)
- Très souvent (toutes les semaines)
- Je ne souhaite pas répondre

*_________________________________________________________________________________*

Avez-vous consulté internet pour le problème médical qui vous a fait appeler la Centrale?

- oui
- non
- je ne souhaite pas répondre

*_________________________________________________________________________________*

Quelle franchise d'assurance maladie avez-vous?

- Je n'ai pas d'assurance maladie
- 300.-
- 500.-
- 1'000.-
- 1'500.-
- 2'000.-
- 2500.-
- Je ne sais pas
- Je ne souhaite pas répondre

*_________________________________________________________________________________*

Quel type d'assurance maladie avez-vous?

- standard
- médecin de famille
- réseau (ex : Delta)
- telmed (medi 24, medgate)
- assurance complémentaire
- Je ne sais pas
- Je ne souhaite pas répondre

*_________________________________________________________________________________*

Nous arrivons au terme des questions que nous souhaitions vous posez. Auriez-vous des commentaires à nous faire suite à votre appel à la Centrale?

- Oui
- Non

*_________________________________________________________________________________*

Précisez

*_________________________________________________________________________________*

**FIN**

*_________________________________________________________________________________*

Commentaires pour la personne qui a fait passer le questionnaire

*_________________________________________________________________________________*
