# Supplementary material for: Impact of a telephone triage service for non-critical emergencies in Switzerland: A cross-sectional study
Source: PLoS One. 2021 Apr 2;16(4):e0249287. doi: 10.1371/journal.pone.0249287 (PMC8018644; doi:10.1371/journal.pone.0249287)
Supplement: S2 File — (DOCX) [file pone.0249287.s002.docx]

**CTMG_enregistrement**

*Veuillez remplir le questionnaire une fois que le questionnaire téléphonique a été complété avec le consentement de participation*.

*Merci !*

_________________________________________________________________________________

Numéro d'identification

_________________________________________________________________________________

Date de l'appel

_________________________________________________________________________________

Jour de la semaine de l'appel

- Lundi
- Mardi
- Mercredi
- Jeudi
- Vendredi
- Samedi
- Dimanche

_________________________________________________________________________________

Jours fériés officiels 2018

Nouvel An : lu. 1er janvier 2018 et ma. 2 janvier 2018

Vendredi Saint : ve. 30 mars 2018

Lundi de Pâques : lu. 2 avril 2018

Jeudi de l'Ascension : je. 10 mai 2018

Lundi de Pentecôte : lu. 21 mai 2018

Fête nationale : me. 1er août 2018

Lundi du Jeûne : lu. 17 septembre 2018

Noël : ma. 25 décembre 2018

Vacances scolaires 2018

Vacances d'hiver : du sa. 23 décembre 2017 au di. 7 janvier 2018

Relâches : du sa. 17 février au di. 25 février 2018

Vacances de Pâques : du ve. 30 mars au di. 15 avril 2018

Pont de l'Ascension : du je. 10 mai au di. 13 mai 2018

Lundi de Pentecôte : lu. 21 mai 2018

Vacances d'été : du sa. 7 juillet au di. 26 août 2018

Jeûne fédéral : lu. 17 septembre 2018

Vacances d'automne : du sa. 13 octobre au di. 28 octobre 2018

Vacances d'hiver : du sa. 22 décembre 2018 au di. 6 janvier 2019

_________________________________________________________________________________

Jour férié?

- Oui
- Non

_________________________________________________________________________________

Période de vacances scolaires?

- Oui
- Non

_________________________________________________________________________________

Heure de l'appel

_________________________________________________________________________________

Code postal de l'appelant

_________________________________________________________________________________

Raison de l'appel

- Baisse de l'état général/je suis pas bien, malaise/perte de connaissance
- Etat grippal/crève/grippe
- Fièvre/température
- Tension trop haute ou trop basse, douleurs dans la poitrine, palpitations/cœur qui bat vite, œdème/jambes gonfles (Problème cardiovasculaire)
- Toux, essoufflement/peine à respirer, bronchite, crachat de sang (Problème respiratoire)
- Nez qui coule, saigne du nez, sinusite, douleurs des oreilles, sifflement dans les oreilles, écoulement des oreilles, diminution de l'ouïe, mal à la gorge, boule dans le cou/adénopathie, vertiges (Problème ORL)
- Mal au ventre, brûlures d'estomac, diarrhée, constipation, sang dans les selles, nausées, vomissements, perte d'appétit, perte/prise de poids, hémorroïdes (Problème abdominal)
- Lumbago/mal au dos, sciatique
- Fracture, entorse, rougeur ou douleur d'une articulation, arthrose (Problème des os ou des articulations autres que le dos)
- Rougeur, brûlure, bouton/éruption, démangeaisons, piqure de tique ou d'insecte, morsure (Problème cutané)
- Hyperglycémie ou hypoglycémie, diabète (Problème métabolique)
- Œil rouge, douloureux, qui coule, démange, vison trouble ou diminuée, corps étranger (Problème oculaire)
- Troubles du sommeil, anxiété, stress, tristesse, idées suicidaires, tentative de suicide (médicamenteux ou autre) (Problème psychologique)
- Migraines/mal à la tête, vertiges, tremblements, perte de force ou de sensibilité d'un membre (Problème neurologique)
- Brûlure en faisant pipi, besoin d'aller uriner plus souvent, sang dans les urines, perte d'urine (Problème urinaire)
- Retard de règle, risque de grossesse, perte de sang, démangeaisons, écoulement anormal/perte (Problème génital)
- Rapport sexuel à risque
- Problème au retour de voyage
- Traumatisme, accident, plaies
- Problème de médicament
- Autre

_________________________________________________________________________________

Précisez:

_________________________________________________________________________________

Qu'est ce qui a été proposé?

- Consulter aux urgences
- Consulter dans une permanence
- Prise de rendez-vous à la maison de la garde
- Mise en contact avec le médecin de garde pour une visite au cabinet
- Mise en contact avec le médecin de garde pour une visite à domicile
- Prendre contact avec son médecin généraliste pour un rendez-vous dès que possible
- Attendre
- Transfert de l'appel au médecin de garde
- Conseils (surveillance, prise de traitement)
- Autre
- Pas d'information

_________________________________________________________________________________

Précisez:

_________________________________________________________________________________

Durée de l'appel

_________________________________________________________________________________
