# Supplementary material for: Impact of a telephone triage service for non-critical emergencies in Switzerland: A cross-sectional study
Source: PLoS One. 2021 Apr 2;16(4):e0249287. doi: 10.1371/journal.pone.0249287 (PMC8018644; doi:10.1371/journal.pone.0249287)
Supplement: S3 File. Questionnaire translated English — (DOCX) [file pone.0249287.s003.docx]

*This is a translation of the original questionnaire, which was in French. The questions to the participants were asked in French.*

**CTMG_Tel**

*The questionnaire has to be completed once the consent form has been approved.*

*Thank you*

----------------------------------------------------------------------------------------------------------------------------------------

*Identification Number:*

----------------------------------------------------------------------------------------------------------------------------------------

*Date:*

----------------------------------------------------------------------------------------------------------------------------------------

*(Info on call at the TTS)*

*Please read the information sheet to the participant and ask him/her if she/he agrees to participate*

We will start by asking you questions about your last call to the telephone triage service (TTS).

----------------------------------------------------------------------------------------------------------------------------------------

Consent to participate given orally:

- Yes
- no

----------------------------------------------------------------------------------------------------------------------------------------

Why did you call the TTS?

- Alteration of the general state / I'm not feeling well, malaise/fainting
- Flu-like symptoms / Influenza
- Fever / temperature
- Blood pressure too high or too low, chest pain, palpitation / heart beating fast, edema/swollen legs (heart disorders)
- Coughing, shortness or difficulty to breath, bronchitis, spitting blood (Breathing disorders)
- Runny nose, nosebleed, sinusitis, ear pain, whistling in the ears, ear discharge, hearing loss, sore throat, lump in the neck/adenopathy, dizziness (ENT disorders)
- Stomach ache, heartburn, diarrhea, constipation, blood in the stools, nausea, vomiting, loss of appetite, loss/gain of appetite/weight, hemorrhoids (Abdominal disorders)
- Back/sciatic pain
- Fracture, sprain, redness or pain of a joint, osteoarthritis (Joint disorders other than the back)
- Redness, burning, pimple/eruption, itching, tick or insect bites, bite (Dermatological disorders)
- Hyperglycemia or hypoglycemia, diabetes (Metabolic disorders)
- Red, painful, runny or itchy eye, diminished or blurred vision, foreign body (ophthalmic disorders)
- Sleep disorders, anxiety, stress, sadness, suicidal ideation, suicide attempts (medical or other) (Psychological disorders)
- Migraines/headaches, dizziness, trembling, loss of strength or feeling in a limb (Neurologic disorders)
- Burning while urinating, need to urinate more often, blood in the urine, loss of urine (Urinary tract disorders)
- Delayed menstruation, risk of pregnancy, loss of blood, itching, abnormal discharge / loss (Genital disorders)
- Risky sexual relation
- Returned traveler questions
- Trauma, accidents, wounds
- Drug-related questions
- Allergy
- Other
- I don't know anymore
- I do not wish to answer

----------------------------------------------------------------------------------------------------------------------------------------

Please specify

----------------------------------------------------------------------------------------------------------------------------------------

If you were unable to call the TTS what would you have done?

- Nothing
- I would have taken medication on my own without consulting a doctor.
- I would have asked a relative (friend, family)
- I would have called my general practitioner as soon as possible
- I would have consulted my general practitioner or another doctor by appointment as soon as possible
- I would have consulted the emergency department
- I would have consulted in a walk-in clinic
- I would have inquired on the internet (or other)
- I would have asked for an opinion from another health professional (osteopath, naturopath, homeopath)
- I would have asked for a pharmacy opinion
- Other
- I don't know
- I do not wish to answer

----------------------------------------------------------------------------------------------------------------------------------------

Please specify

----------------------------------------------------------------------------------------------------------------------------------------

At the time of your call, what kind of care have you been offered as next steps?

- Consult the emergency department
- Consulting in a walk-in clinic
- Making an appointment at the “maison de la garde”
- Contact with the physician on duty
- Contact my general practitioner for an appointment as soon as possible
- Waiting
- Transfer of the call to the physician on duty for advice on further treatment
- Advice (monitoring, temperature control, treatment intake)
- Using another service
- Other
- I don't know
- I do not wish to answer

----------------------------------------------------------------------------------------------------------------------------------------

Please specify

----------------------------------------------------------------------------------------------------------------------------------------

What did you do after your call with the TTS?

- I called 144 for an ambulance.
- I went to the emergency department
- I've consulted in a walk-in clinic
- I went to the appointment at the at “maison de la garde”
- I had contact with the physician on duty for a visit to the office
- I had contact with the physician on duty for a home visit
- I contacted my general practitioner for an appointment as soon as possible.
- I have waited
- I followed the advice (monitoring etc.)
- I have called back the TTS
- Other
- I don't know
- I do not wish to answer

----------------------------------------------------------------------------------------------------------------------------------------

Please specify

----------------------------------------------------------------------------------------------------------------------------------------

Why did you call the TTS instead of another service?

- I like to call the TTS
- It was out of office hours
- I was too sick to go out
- I have followed the advice of friends/family
- I didn't know who to contact
- It was urgent / I didn't want to wait
- I didn't want to bother my general practitioner
- I was anxious
- My general practitioner was not available
- I don't have a general practitioner
- On the advice of my health insurance
- Other
- I don't know
- I do not wish to answer

----------------------------------------------------------------------------------------------------------------------------------------

Please specify

----------------------------------------------------------------------------------------------------------------------------------------

How do you know the TTS?

- Via an acquaintance (friend, family, close friend)
- By my general practitioner
- By my pharmacist
- By another health professional
- Through the media
- Other
- I don't know
- I do not wish to answer

----------------------------------------------------------------------------------------------------------------------------------------

Please specify

----------------------------------------------------------------------------------------------------------------------------------------

Was this your first call to the TTS?

- Yes
- No

----------------------------------------------------------------------------------------------------------------------------------------

If not, approximately how many calls did you make for yourself in the last 12 months?

- this was the first call in the last 12 months
- 2-5
- 5-10
- More than 10

----------------------------------------------------------------------------------------------------------------------------------------

**We are going to ask you some questions about your satisfaction following the call**

----------------------------------------------------------------------------------------------------------------------------------------

Overall, on a scale of zero to ten, how would you rate your satisfaction with the call to the TTS? (0=not at all satisfied, 10=very satisfied)

On a scale of zero to ten, how well do you did you feel listened to by the person who has answered your call?
(0=not at all listened to, 10=fully listened to)

Always about your call, on a scale of zero to ten, at what point would you say that the call has met your expectations?
(0=not at all, 10=full)

On a scale of zero to ten, how would you rate the professionalism of the charge of your call?
(0=not at all professional, 10=very professional)

----------------------------------------------------------------------------------------------------------------------------------------

How long do you think you waited for an answer?

- Less than a minute
- 1 to 5 minutes
- 6 to 10 minutes
- 11 to 15 minutes
- 16 to 30 minutes
- 31 minutes to 1 hour
- more than one hour
- I don't know
- I do not wish to answer

----------------------------------------------------------------------------------------------------------------------------------------

**Demography**

----------------------------------------------------------------------------------------------------------------------------------------

We will now ask some general questions about you as a person.

----------------------------------------------------------------------------------------------------------------------------------------

What is your gender?

- Woman - man – other

----------------------------------------------------------------------------------------------------------------------------------------

How old are you? (in years)

----------------------------------------------------------------------------------------------------------------------------------------

What is your marital status?

- Single
- Married
- Widowed
- Divorced
- Separated
- Linked by registered partnership / Living together
- Registered partnership dissolved
- Other

----------------------------------------------------------------------------------------------------------------------------------------

In which country were you born?

- Switzerland
- Eastern Europe (Russia, Ukraine, Belarus)
- Western Europe (United Kingdom, France, Ireland, Belgium, Netherlands, Luxembourg)
- Northern Europe (Norway, Denmark, Iceland, Sweden, Finland, Estonia)
- Southern Europe (Spain, Portugal, Italy, Sint Maarten, Croatia, Greece, Montenegro, Bulgaria, Bosnia, Serbia, Albania, Macedonia, Kosovo)
- North America
- South America
- North Africa
- South Africa
- Asia
- Other

----------------------------------------------------------------------------------------------------------------------------------------

Please specify

----------------------------------------------------------------------------------------------------------------------------------------

How long have you lived in Switzerland?

- less than one year
- more than one year
- I do not wish to answer

----------------------------------------------------------------------------------------------------------------------------------------

Please specify

----------------------------------------------------------------------------------------------------------------------------------------

Which passport or residence permit do you have?

- Swiss passport
- residence permit
- tourist or student
- other
- I don't know
- I do not wish to answer

----------------------------------------------------------------------------------------------------------------------------------------

What is the highest level of education you have completed?

- has not completed or is still attending compulsory school
- completed compulsory school
- apprenticeship, vocational school
- high school diploma
- university
- other
- I don't know
- I do not wish to answer

----------------------------------------------------------------------------------------------------------------------------------------

Please specify

----------------------------------------------------------------------------------------------------------------------------------------

How old were you when you left school?

----------------------------------------------------------------------------------------------------------------------------------------

Do you have children?

- Yes
- No

----------------------------------------------------------------------------------------------------------------------------------------

If yes, how many?

----------------------------------------------------------------------------------------------------------------------------------------

Are they dependent children?

- yes all
- yes some of them
- no

----------------------------------------------------------------------------------------------------------------------------------------

Please indicate the age of the child?
(in years, if less than 1 year old: 0.1 for 1 month, o.2 etc up to 0.11 for 11 months)

----------------------------------------------------------------------------------------------------------------------------------------

Please indicate the age of the child?

----------------------------------------------------------------------------------------------------------------------------------------

Please indicate the age of the child?

----------------------------------------------------------------------------------------------------------------------------------------

Please indicate the age of the child?

----------------------------------------------------------------------------------------------------------------------------------------

Please indicate the age of the child?

----------------------------------------------------------------------------------------------------------------------------------------

Please indicate the age of the child?

----------------------------------------------------------------------------------------------------------------------------------------

Please indicate the age of the child?

----------------------------------------------------------------------------------------------------------------------------------------

How many of them live at home?

----------------------------------------------------------------------------------------------------------------------------------------

What is the structure of your household and the place you live?

- Living alone
- Living with parents or one parent
- Living as a couple without children
- Lives as a couple with child(ren)
- Parent raising one or more children alone
- Flat sharing with friends, acquaintances
- In a student residence, boarding school
- In a social institution (social residence, medico-social institution)
- Homeless
- Other
- I do not wish to answer

----------------------------------------------------------------------------------------------------------------------------------------

Do you currently have a professional activity?

- no
- yes part-time
- yes full time

----------------------------------------------------------------------------------------------------------------------------------------

Are you currently receiving a pension?

- No
- Unemployment compensation
- Social pension
- Disability insurance pension
- Old-age and survivors' insurance pension
- Other
- I don't know
- I do not wish to answer

----------------------------------------------------------------------------------------------------------------------------------------

**State of health**

----------------------------------------------------------------------------------------------------------------------------------------

We would now like to ask you some questions about your state of health.

----------------------------------------------------------------------------------------------------------------------------------------

How would you rate your general health?

- Very good
- good
- fairly good
- bad
- very bad
- I do not wish to answer

----------------------------------------------------------------------------------------------------------------------------------------

Do you have a general practitioner?

- Yes
- No

----------------------------------------------------------------------------------------------------------------------------------------

If yes, where?

- in a practice
- in a walk-in clinic

----------------------------------------------------------------------------------------------------------------------------------------

Please indicate if you currently suffer from any of the following illnesses:

- None
- High blood pressure
- Heart disorders
- Diabetes
- Chronic bronchitis (COPD)
- Osteoarthrosis/joint disorders
- Other pain
- Cancer
- Other
- I do not wish to answer

----------------------------------------------------------------------------------------------------------------------------------------

Please specify

----------------------------------------------------------------------------------------------------------------------------------------

Are you currently taking any medication every day for several months?

*--> (we are trying to find out if chronic treatment, exclusion of food supplements, vitamins, homeopathy, Bach drops and other complementary medicine)*

*--> for women think about asking if taking contraception*

- Yes
- No

----------------------------------------------------------------------------------------------------------------------------------------

On average, how many treatment/medication do you take every day?

*(we are trying to find out the number of different medicines per day and not if taking 1 medication several times a day)*

----------------------------------------------------------------------------------------------------------------------------------------

In the past 12 months, have you consulted one of the following health professionals at least once? If yes, which one(s)?

- Family doctor, general practitioner, internist
- Specialist doctor
- Other health professional (diabetes nurse, dietician, chiropodist, psychologist, physiotherapist)
- Hospital Emergency Department
- Walk-in clinic
- Other
- None

----------------------------------------------------------------------------------------------------------------------------------------

How many times in the last 12 months?

----------------------------------------------------------------------------------------------------------------------------------------

Please specify

----------------------------------------------------------------------------------------------------------------------------------------

Do you ever use the internet to find out about medical disorders ?

- Yes
- no
- I do not wish to answer

----------------------------------------------------------------------------------------------------------------------------------------

If yes, how often do you use the internet for health information?

- Never
- Almost never (less than once a year)
- Sometimes (1-3x per year)
- Often (every month)
- Very often (every week)
- I do not wish to answer

----------------------------------------------------------------------------------------------------------------------------------------

Did you consult the internet for the medical problem that lead you to call the TTS?

- Yes
- No
- I do not wish to answer

----------------------------------------------------------------------------------------------------------------------------------------

What fixed annual amount of the health insurance do you have?

- I do not have any health insurance
- 300.-
- 500.-
- 1'000.-
- 1'500.-
- 2'000.-
- 2500.-
- I don't know
- I do not wish to answer

----------------------------------------------------------------------------------------------------------------------------------------

What type of health insurance do you have?

- standard
- general practitioner
- network (e.g. Delta)
- telmed (medi 24, medgate)
- complementary insurance
- I don't know
- I do not wish to answer

----------------------------------------------------------------------------------------------------------------------------------------

We are coming to the end of the questions we wanted to ask you. Do you have any comments you would like to make following your call to the Central?

- Yes
- No

----------------------------------------------------------------------------------------------------------------------------------------

Please specify

----------------------------------------------------------------------------------------------------------------------------------------

**End**

----------------------------------------------------------------------------------------------------------------------------------------

Comments for the person who administered the questionnaire

----------------------------------------------------------------------------------------------------------------------------------------

**CTMG_enregistrement**

*Please complete the questionnaire once the telephone questionnaire has been completed with the consent of participation.*

Thank you

----------------------------------------------------------------------------------------------------------------------------------------

Identification number

----------------------------------------------------------------------------------------------------------------------------------------

Date of the call

----------------------------------------------------------------------------------------------------------------------------------------

Day of the week of the call

- Monday
- Tuesday
- Wednesday
- Thursday
- Friday
- Saturday
- Sunday

----------------------------------------------------------------------------------------------------------------------------------------

Official public holidays 2018

New Year's Day: Mon. 1 January 2018 and Tue. 2 January 2018
Good Friday: Fri. 30 March 2018
Easter Monday: Mon 2 April 2018
Ascension Thursday: I. 10 May 2018
Whit Monday: Mon. 21 May 2018
National holiday: 1st August 2018
Fasting Monday: Mon. 17 September 2018
Christmas: May 25, 2018

School holidays 2018

Winter holidays: from Sat. 23 December 2017 to Sun. 7 January 2018
Breaks: from Sat. 17 February to Sun. 25 February 2018
Easter Holidays: Fri. 30 March to Sun. 15 April 2018
Ascension: from 10 May to 13 May 2018
Whit Monday: Mon. 21 May 2018
Summer holidays: Sat. 7 July to Sun. 26 August 2018
Federal fast: Mon. 17 September 2018
Autumn holidays: from Sat. 13 October to Sun. 28 October 2018
Winter holidays: from Sat. 22 December 2018 to Sun. 6 January 2019

----------------------------------------------------------------------------------------------------------------------------------------

Public holiday?

- Yes
- No

----------------------------------------------------------------------------------------------------------------------------------------

School holidays?

- Yes
- No

----------------------------------------------------------------------------------------------------------------------------------------

Time of call

----------------------------------------------------------------------------------------------------------------------------------------

Caller's ZIP code

----------------------------------------------------------------------------------------------------------------------------------------

Reasons of the call

- Alteration of the general state / I'm not feeling well, malaise/fainting
- Flu-like symptoms / Influenza
- Fever / temperature
- Blood pressure too high or too low, chest pain, palpitation / heart beating fast, oedema/swollen legs (heart disorders)
- Coughing, shortness or difficulty to breath, bronchitis, spitting blood (Breathing disorders)
- Runny nose, nosebleed, sinusitis, ear pain, whistling in the ears, ear discharge, hearing loss, sore throat, lump in the neck/adenopathy, dizziness (ENT disorders)
- Stomach ache, heartburn, diarrhea, constipation, blood in the stools, nausea, vomiting, loss of appetite, loss/gain of appetite/weight, hemorrhoids (Abdominal disorders)
- Back/sciatic pain
- Fracture, sprain, redness or pain of a joint, osteoarthritis (Joint disorders other than the back)
- Redness, burning, pimple/eruption, itching, tick or insect bites, bite (Dermatological disorders)
- Hyperglycemia or hypoglycemia, diabetes (Metabolic disorders)
- Red, painful, runny or itchy eye, diminished or blurred vision, foreign body (ophthalmic disorders)
- Sleep disorders, anxiety, stress, sadness, suicidal ideation, suicide attempts (medical or other) (Psychological disorders)
- Migraines/headaches, dizziness, trembling, loss of strength or feeling in a limb (Neurologic disorders)
- Burning while urinating, need to urinate more often, blood in the urine, loss of urine (Urinary tract disorders)
- Delayed menstruation, risk of pregnancy, loss of blood, itching, abnormal discharge / loss (Genital disorders)
- Risky sexual relation
- Returned traveler questions
- Trauma, accidents, wounds
- Drug-related questions
- Other

----------------------------------------------------------------------------------------------------------------------------------------

Please specify

----------------------------------------------------------------------------------------------------------------------------------------

What has been proposed?

- Consult the emergency department
- Consulting in a walk-in clinic
- Making an appointment at the “maison de la garde”
- Contact with the physician on duty
- Contact my general practitioner for an appointment as soon as possible
- Waiting
- Transfer of the call to the physician on duty for advice on further treatment
- Advice (monitoring, temperature control, treatment intake)
- Using another service
- Other
- No information

----------------------------------------------------------------------------------------------------------------------------------------

Please specify

----------------------------------------------------------------------------------------------------------------------------------------

Duration of the call

----------------------------------------------------------------------------------------------------------------------------------------
